# Natural Zeolite Clinoptilolite Application in Wastewater Treatment: Methylene Blue, Zinc and Cadmium Abatement Tests and Kinetic Studies

**DOI:** 10.3390/ma15228191

**Published:** 2022-11-18

**Authors:** Melodj Dosa, Nadia Grifasi, Camilla Galletti, Debora Fino, Marco Piumetti

**Affiliations:** Department of Applied Science and Technology, Corso Duca Degli Abruzzi, 24, 10129 Turin, Italy

**Keywords:** Clinoptilolite, Wastewater Treatment, Adsorption, Kinetic, Methylene Blue, Metal Cations

## Abstract

In recent decades, several abatement techniques have been proposed for organic dyes and metal cations. In this scenario, adsorption is the most known and studied. Clinoptilolite was considered, since it is a zeolite with a relatively low cost (200–600 $ tons^−1^) compared to the most well-known adsorbent used in wastewater treatment. In this work, Clinoptilolite was used for the adsorption of Methylene Blue (MB) at three different concentrations, namely, 100, 200, and 250 ppm. Furthermore, the adsorption capacity of the natural zeolite was compared with that of Activated Charcoal (250 ppm of MB). The two adsorbents were characterized by complementary techniques, such as N_2_ physisorption at −196 °C, X-ray diffraction, and field emission scanning electron microscopy. During the adsorption tests, Clinoptilolite exhibited the best adsorption capacities at 100 ppm: the abatement reached 98% (t = 15 min). Both Clinoptilolite and Activated Charcoal, at 250 ppm, exhibited the same adsorption capacities, namely, 96%. Finally, at 250 ppm MB, the adsorption capacity of Clinoptilolite was analyzed with the copresence of Zn^2+^ and Cd^2+^ (10 ppm), and the adsorption capacities were compared with those of Activated Charcoal. The results showed that both adsorbents achieved 100% MB abatement (t = 40 min). However, cation adsorption reached a plateau after 120 min (Zn^2+^ = 86% and 57%; Cd^2+^ = 53% and 50%, for Activated Charcoal and Clinoptilolite, respectively) due to the preferential adsorption of MB molecules. Furthermore, kinetic studies were performed to fully investigate the adsorption mechanism. It was evidenced that the pseudo-second-order kinetic model is effective in describing the adsorption mechanism of both adsorbents, highlighting the chemical interaction between the adsorbent and adsorbate.

## 1. Introduction

During recent decades, several abatement techniques have been proposed for metals and organic compounds in wastewater treatment [1,2,3,4,5,6]. Łach et al. [6] proposed an innovative method to modify synthetic zeolites with calcium to increase their cation exchange capability for potential adsorption processes.

To facilitate the abatement of dyes, Adhikari et al. [1] proposed hydrogels based on synthetic self-assembling tripeptides. McMullan et al. [2] and Pearce et al. [3] proposed microbes and bacteria, respectively, since they can decolorize and metabolize dyes. Dosa et al. [4], Piumetti et al. [7] and Freyria et al. [8] demonstrated the effect of natural and modified zeolites, titania and titania-doped materials on the abatement of Acid Orange 7.

Dyes are commonly used in the textile, paper, and cosmetics industries [1,2,3,9,10], and they are difficult to abate since they are stable in light, heat, and oxidant environments [11,12]. Among the organic dyes, Methylene Blue (C_16_H_18_N_3_SCl) is widely studied [13,14,15,16]. Specifically, Methylene Blue (herein labeled “MB”) at higher concentrations can generate several health issues in humans [17] and exhibits a toxic effect on aquatic life [18]. The works of EL-Mekkawi et al. [19], Mulushewa et al. [20], Dosa et al. [14] and Galletti et al. [21] evidenced that naturally derived adsorbents, such as kaolin, grape-wood wastes or Clinoptilolite, exhibited interesting effects on MB. On the other hand, the adsorbent material derived from laboratory synthesis, such as activated carbon or mesoporous silica, exhibited good results toward the same pollutant, as reported by Mousavi et al. [22] and Nicola et al. [23], respectively.

In terms of metal adsorption, Hussain et al. [5] demonstrated that Activated Charcoal, zeolite, clay minerals, and nanostructured materials can be widely used for this application. Interesting outcomes can also be seen in the study of Galletti et al. [21], which demonstrated that the natural zeolite Clinoptilolite can be used for the abatement of metal cations. Some of the heavy metal cation groups are micronutrients for the biological functions of organisms [24,25]. However, when the amount of such metals is higher than the minimum values, several problems can occur in the biological functions [5,24,25,26,27,28]. Among the heavy metal pollutants, Zn^2+^ and Cd^2+^ are of particular interest, and their limits are fixed for different kinds of wastewater discharges [29,30,31]. Over the years, several techniques have been developed for the abatement of metal cations in wastewater, such as the ion exchange method by O’Connel et al. [32] or chemical precipitation by Hunsom et al. [33]. There are other interesting techniques that, for the sake of brevity, have not been mentioned [34,35]. In this scenario, the adsorption toward the metal cation can be considered a good choice since good results were presented in the study of Bobade and Eshtiaghi [36].

Since zeolites are used for the abatement of organic dyes and metal cations, in this study, Clinoptilolite, a natural zeolite, was considered. The reason for such interest is not only due to its potential applications but also due to its cost, which is cheaper (100–600 $ tons^−1^) compared to synthetic zeolites [37]. Clinoptilolite belongs to the HEU family-type zeolite [38], and it is a zeolite that occurs naturally. Thus, its chemical composition changes depending on the geographical area of the extraction [39,40,41]. As a whole, the large variety of metal cations inside its three-dimensional tectosilicate can help during the abatement or degradation processes toward specific contaminants, as observed in other research works [14,21,42,43,44,45,46,47,48].

In this work, Clinoptilolite (named “Clin” from now on) was studied for Methylene Blue, Zn^2+^, and Cd^2+^ abatement tests. Activated Charcoal, labeled “AC”, was investigated for comparison purposes. MB was considered a probe molecule for organic pollutants, while zinc and cadmium cations were considered for heavy metal contamination.

Moreover, a kinetic study was conducted to better understand the mechanisms that may influence the adsorption process, and a model suitable for the examined systems was proposed that better describes the adsorbate–adsorbent interactions. The aim is to predict the rate at which contaminants are removed from aqueous solutions to design and optimize appropriate adsorption-based water treatment operations [49,50,51,52,53,54,55].

## 2. Materials and Methods

### 2.1. Adsorbent Materials

The adsorbent materials studied in this work were the natural zeolite Clin (Zeolado, Greece) and AC (Norit ROW 0.8 Supra, Merk, Steinheim, Germany). The Clinoptilolite used is characterized by a sedimentary origin (≥90%) and appears as a light gray–green solid powder with a specific weight of approximately 2200–2440 kg m^−3^. The AC comprises black granules with an average dimension of particles (≤0.5%) less than 0.60 mm. The water content, evaluated by the Karl Fisher Titration method, was lower than 0.5%.

### 2.2. Structural and Textural Characterizations

The crystalline structures of Clin and AC were investigated using powder X-ray diffraction analysis (XRD). The patterns were collected with an X’Pert Philips PW3040 (Malvern Panalytical Ltd., Malvern, UK) diffractometer using Cu K*_α_* radiation (2θ range = 5°–50°; step = 0.05° 2θ; time per step = 0.2 s). Then, the collected diffraction peaks were indexed according to the Powder Data File database (PDF-2 1999, International Centre of Diffraction Data).

N_2_ physisorption at −196 °C was performed to evaluate the Specific Surface Area (SSA, m^2^ g^−1^) and the Total Pore Volume (V_TP_, cm^3^ g^−1^) of the adsorbent materials in a Micromeritics Tristar II 3020 (v1.03, Micromeritics Instrument Corp., Norcross, GA, USA). The SSA was evaluated using the Brunauer–Emmett–Teller (BET) method, and the V_TP_ was evaluated using the Barrett–Joyner–Halenda (BJH) method during the desorption phase. Before the analysis, the samples were pretreated under N_2_ flow at 200 °C for 4 h to eliminate impurities on their surface (i.e., water).

The morphology of the samples and the elemental composition (EDX analysis) were studied through field emission scanning electron microscopy analysis (FESEM, Zeiss MERLIN, Gemini-II column, Oberkochen, Germany). X-ray fluorescence analysis was performed with a RIGAKU ZSX100E apparatus (Tokyo, Japan) equipped with an Rh X-ray tube and TAP, PET, LiF1, Ge, RX61, and RX45 analysis crystals. Samples were prepared by pressing powder into thin discs with a diameter of 20 mm and a thickness of 2 mm. The samples were analyzed at least 20 different points.

### 2.3. Abatement Tests

#### 2.3.1. Methylene Blue Abatement with Clinoptilolite

MB (Merck, Steinheim, Germany) was chosen as a probe molecule for the organic pollutant. The concentration of MB for the abatement tests was 100, 200, and 250 ppm. The choice of these MB concentrations was taken after a literature study. Several works present tests at lower (10–110 ppm [56,57]) or higher (200–1000 ppm [58,59]) concentrations of MB. If the tests were performed with lower MB concentrations, the adsorption proceeds too fast, and it is difficult to properly discriminate the kinetics of the adsorption. On the other hand, with a higher amount, namely, higher than 250 ppm, saturation of the active sites of the adsorbent materials could occur. For this reason, 100, 200, and 250 ppm concentrations were chosen. The MB solution was put into a beaker and placed on a magnetic stirrer. The adsorbent material, Clin, (5 g L^−1^) was placed inside the MB solution. The abatement test started when Clin was added to the solution (time 0), and then a small amount of solution was collected over time, centrifuged, and subsequently analyzed with a UV–VIS spectroscope (HACH LANGE GmbH, Ames, Iowa, USA) using 664 nm as the absorbance reference peak of MB.

#### 2.3.2. Methylene Blue Abatement with Activated Charcoal

The MB abatement tests with AC were performed with a 250 ppm dye concentration. The AC concentration was 10 g L^−1^. The tests were performed with the same procedure described in MB abatement with Clin (Section 2.3.1). For this case study, the adsorption capacity toward AC was compared with Clin at the same concentration.

#### 2.3.3. Abatement Tests with Methylene Blue and Metal Cations

Finally, abatement tests were performed with the presence of MB, Cd^2+^, and Zn^2+^ at 250 ppm, 10 ppm, and 10 ppm, respectively. The Clin and AC concentrations were 10 g L^−1^. The collected suspension was analyzed via a UV–Vis spectrometer (MB) and ICP (iCAP Q ICP–MS, ThermoFisher Scientific, Waltham, MA, USA) for metal concentrations. The tests were performed with the same procedure described in MB abatement with Clin (Section 2.3.1).

## 3. Results

### 3.1. Structural and Textural Properties

Table 1 reports the results as derived from the N_2_ physisorption at −196 °C. The AC exhibited higher textural properties (SSA = 891 m^2^ g^−1^, V_TP_ = 0.56 cm^3^ g^−1^) than Clin (SSA = 32 m^2^ g^−1^, V_TP_ = 0.12 cm^3^ g^−1^). The results of the SSA and V_TP_ of Clin are in agreement with other literature results [4,21].

**Figure 1:**
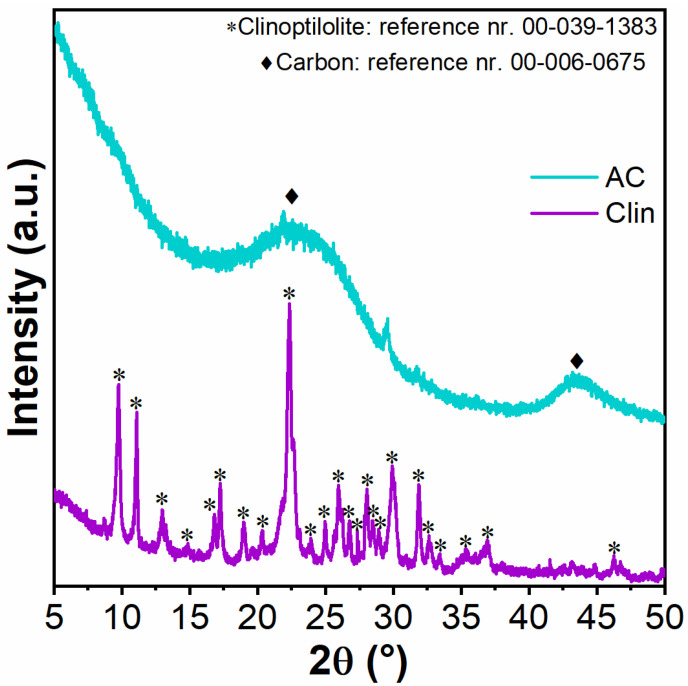
X-ray diffractograms in the range 5° < 2θ < 50° over Clin and AC.

The crystallinity of Clin and AC was investigated using the X-ray diffraction technique. The results are reported in Figure 1. Clin exhibits high crystallinity, and the most intense peaks are at 2θ = 9.92°, 11.09°, and 22.43°, according to the reference pattern in the database (00-039-1383) and other previous studies [4,14,21]. The peak at 2θ = 22.43° is the most intense peak of the natural zeolite and is denoted by the (1 3 1)-type plane [61]. Davarpanah et al. [47] demonstrated that Clin has a different percentage of minerals, reported in Table S1, comprising an amorphous phase, kaolinite, and illite, and the rest are Clinoptilolite minerals.

On the other hand, AC exhibits a low crystallinity pattern, as observed in the literature [62,63]. The two peaks at 22.56° and 43.57° are the (0 0 2) and (1 0 1)-type planes of the typical charcoal pattern [62]. The AC diffractogram was compared with reference pattern 00-006-0675.

**Figure 2:**
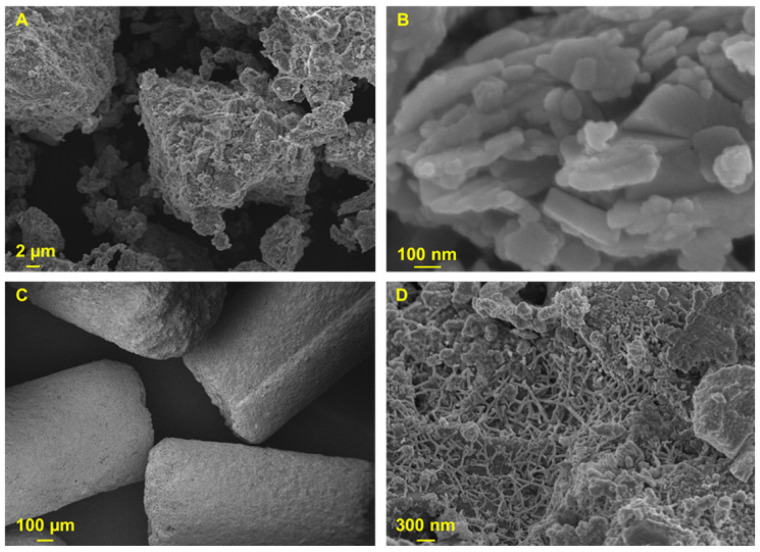
FESEM images of Clin (**A**,**B**) and AC (**C**,**D**) at different magnifications.

The morphology of the samples was investigated with FESEM micrography, and the results are reported in Figure 2. Clin exhibits the typical flake-like structure confirmed by FESEM analysis (Figure 2A,B) [4,14,21,39,40,64]. On the other hand, AC presents a multiwallet mesopore structure [62,63], as reported in Figure 2C,D.

**Figure 3:**
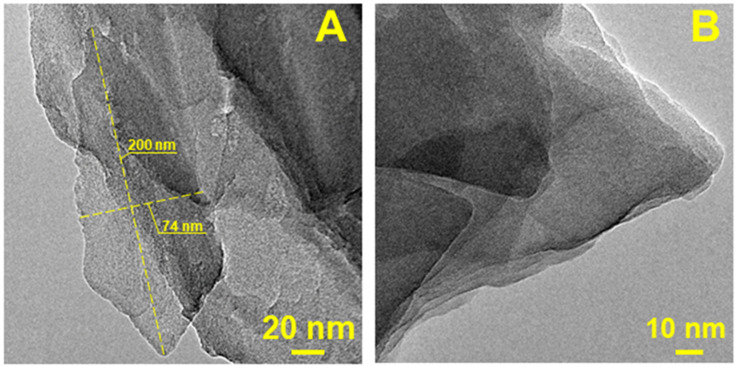
TEM images of Clin at two different magnifications, (**A**) 20 nm and (**B**) 10 nm.

Figure 3 shows two different magnifications of Clin TEM images. It is possible to demonstrate the flake-like structure of Clin. Moreover, in Figure 3A, it was possible to evaluate the average dimension of a particular Clin particle, which is approximately 200 × 74 nm, using ImageJ software [65].

The chemical composition of Clin was investigated by EDX and XRF analysis; the results are reported in Table 2.

The Si-to-Al ratio was approximately 5 from EDX analysis and 7 from the XRF results. As demonstrated from the results reported in Table 2, the values obtained from the two above-mentioned techniques are quite similar to each other and the differences are not significant.

### 3.2. Abatement Tests

#### 3.2.1. Methylene Blue Abatement with Clinoptilolite and Activated Charcoal

As evidenced by the literature, MB adsorption proceeds through four steps [66]: (a) migration of MB from the bulk solution to the surface of the adsorbent; (b) MB diffusion in the boundary layer to the surface of the adsorbent; (c) MB adsorption on the active sites of the material; and (d) intraparticle diffusion of MB into material pores. In addition, MB adsorption can be influenced by the pH of the solution. This parameter influences the surface charge of Clin, the degree of ionization, and the electrical charge of MB [67]. In this work, the pH was not adjusted to perform the test in a more realistic situation.

In an aqueous solution, when the pH value is high, the H^+^ concentration is very low, and a strong interaction originates from the Clin surface and MB cation molecule. The interaction between Clin and MB at high pH values is possible only due to the ion-exchange properties of Clin. Since Clin is a zeolite, its surface has negative charges, which are compensated by the presence of metal cations on the surface. The presence of several different metals on the Clin surface was demonstrated by EDX and XRF measurements (see Table 2 Section 3.1). In solution, MB is a cationic molecule. On the other hand, Clin in solution can easily exchange its metal cation on the surface, and as a consequence, the Clin surface exhibits negative charges, which are considered active sites during the adsorption process. If Clin can easily exchange its metal cations on the surface, it is possible to create a new strong interaction, Clin-MB [21,68,69,70], because the cationic MB is strongly attracted by the negative active sites on the Clin surface. As a consequence, the interaction of Clin-MB increases the adsorption capacity of the material [67].

**Figure 4:**
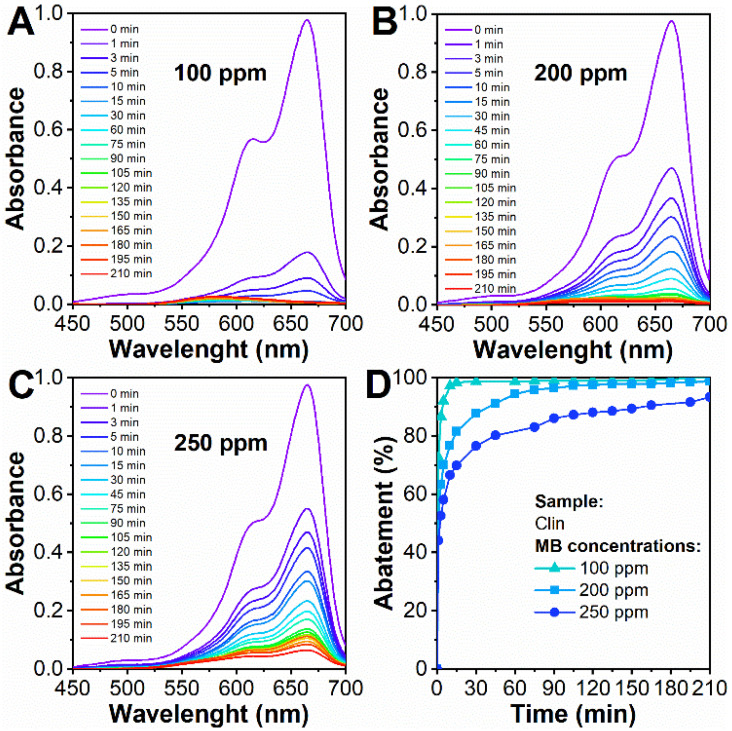
Clin (5 g L^−1^) abatement tests were performed with 100 ppm (**A**), 200 ppm (**B**), and 250 ppm (**C**) MB concentrations. MB abatement as a function of time (**D**) at three concentrations, 100, 200, and 250 ppm.

The results of the MB abatement tests with Clin are reported in Figure 4. All the absorbance spectra, in Figure 4, were normalized. To further investigate the behavior of Clin toward MB, Figure S1 shows the adsorption isotherm constructed from different concentrations of organic dye, the form of which can be related to an L-type.

For the sake of completeness, the amount of MB adsorbed over time on Clin is shown in Figure S2. The pH of the MB solution was measured at the beginning and at the end of the tests, and the pH measurements are reported in Table 3.

As expected, when the MB concentration is 100 ppm (Figure 4A), the abatement proceeds more efficiently compared to higher concentrations, namely, 200 and 250 ppm (Figure 4B,C). As a whole, Clin was able to capture 100, 99, and 93% of MB with 100, 200, and 250 ppm case studies, respectively (adsorption time = 210 min). The behavior and abatement kinetics for these three case studies will be examined in Section 3.3.

Zeolites are materials that easily adsorb water molecules in comparison to dyes in an aqueous solution. Thus, the active sites of Clin start to become inaccessible to MB molecules when the amount of dye increases. However, thanks to the Clin structure as well as its unique chemical composition, which is able to create negative charges on the Clin surface after ion exchange, this natural zeolite has good abatement capacities for removing cationic dyes such as MB [67,71].

As mentioned before, the abatement process is influenced by the pH of the solution [67]. The lower the MB concentration was, the higher the starting pH value (Table 3).

In fact, the best abatement condition is represented for the 100 ppm case study due to the effect of the initial pH value (Figure 4A). When the MB amount increases, the pH decreases since the OH- species in solution are attracted to cationic MB, and the pH decreases to 8.01 and 6.18 at 200 and 250 ppm, time zero min, respectively. As a consequence, if the amount of MB increases, the active sites are saturated immediately since their number does not change during the process. For this reason, at higher concentrations, namely, 200 and 250 ppm, the kinetics proceed slowly, and MB abatement is not performed as in the 100 ppm case study.

**Figure 5:**
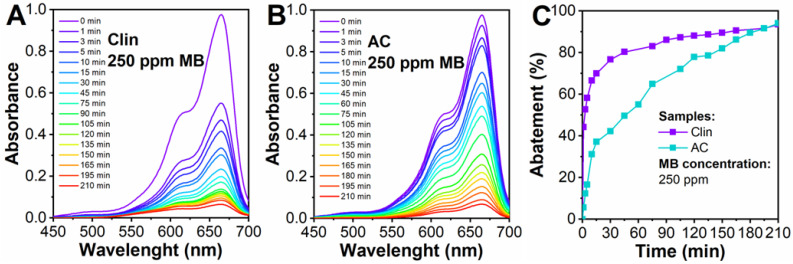
Clin (5 g L^−1^) abatement tests (**A**) and AC (5 g L^−1^) (**B**) were performed with 250 ppm MB. MB abatement as a function of time (**C**) at 250 ppm MB.

Figure 5 reports the comparison between Clin and AC in terms of MB abatement with the highest concentration (250 ppm) to better discriminate their adsorption capacities. Additionally, for this case study, the absorbance spectra were normalized. Instead, Figure S3 shows the amount of MB adsorbed over time comparing the two adsorbents.

The reason for the comparison between Clin and AC is to perform an analysis of possible alternatives to AC, which is a material commonly used for wastewater treatment. As previously shown, Clin has an average price of approximately 200–600 $ tons^−1^ [37]. On the other hand, the AC has a price that changes depending on the procedure and the matrix used [72,73,74,75]. For detailed costs of AC materials, the information is reported in Table S2. Overall, the costs of AC are approximately 1.08–2.89 $ kg^−1^. Thus, it is evident that Clin is cheaper than AC, and the aim is to investigate the adsorption of such contaminants with a valid and low-cost alternative.

As a whole, the MB abatement with Clin (Figure 5A) proceeds faster compared to AC (Figure 5B). The reason for such behavior could be explained by the abundant presence of cations in the Clin framework. As exposed above, the presence of metal cations on the Clin surface as well as the good ion-exchange properties of this natural zeolite can create a strong MB–Clin interaction [21,68,69,70]. The MB can be easily attracted on the Clin surface, and the abatement proceeds faster compared with the AC case study. As reported by Figueiredo and Pereira [76] the active sites in AC can be carboxyl, lactone, lactol, phenol, carbonyl, anhydride, ether, quinone, pyrone, chromene, pyridine, quaternary, pyridine, oxidized N, and pyrrole groups. Despite a variety of active sites, it is evident from the results reported in Figure 5 that the interaction of the cationic MB in solution with the active sites of Clin is stronger than the possibility of having an interaction with such a variety of active sites on AC. The reason could be the partial hydration of such chemical groups in water, which does not allow a strong interaction with cationic MB.

It is interesting to observe that at the end of the abatement tests, namely, 210 min adsorption time, both materials reached approximately 94% MB abatement (Figure 5C). Although AC does not have metal cations on the surface, its higher surface area is able to store a high quantity of MB for longer adsorption times. Steps (c) and (d) of the MB adsorptions are more optimized on AC compared to Clin. First, a high surface area implies the presence of more active sites (step 3 of MB adsorption: the higher the number of active sites is, the higher the amount of MB adsorbed). Furthermore, the AC pores are larger compared to Clin, which is a zeolite with micropores, and the AC does not have intradiffusional problems like Clin.

As is evident from the comparison with the literature (see Table S3 in the Supporting Information), the results achieved are comparable to those obtained in a previous work by Dosa et al. [14] for the case study of 250 ppm of MB, but seem much more promising compared to other studies in which, on the one hand, 100% abatement of the organic dye was not achieved [15] and, on the other hand, the time required to achieve appreciable abatement is extremely long, as described in the study by Sun et al. [12]. These limits were exceeded in the present work, e.g., 100% abatement of the dye pollutant was achieved after approximately 30 min at a concentration of 100 ppm.

#### 3.2.2. Methylene Blue, Zn^2+^ and Cd^2+^ Abatement with Clinoptilolite and Activated Charcoal

**Figure 6:**
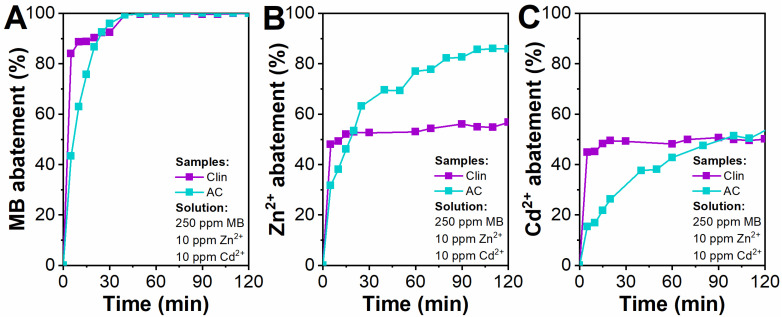
Clin and AC abatement tests (10 g L^−1^) with 250 ppm MB, 10 ppm Zn^2+^, and 10 ppm Cd^2+^. Abatement of MB (**A**), Zn^2+^ (**B**), and Cd^2+^ (**C**) as a function of time.

Finally, the effect of the copresence of MB, Zn^2+^, and Cd^2+^ for the Clin and AC case study was analyzed. The results are reported in Figure 6.

As a whole, both Clin and AC exhibit promising abatement capacities toward the MB molecule since the MB abatement is above 90% after 30 min (Figure 6A).

For Zn^2+^ and Cd^2+^, the abatement does not reach 100% for both adsorbent materials (Figure 6B and Figure 6C, respectively). The reason for such behavior could be explained by the preferential adsorption of MB on the Clin surface. It was observed that Clin does not prefer the adsorption of Zn^2+^ instead of Cd^2+^, which is in agreement with a previous study. The adsorption of divalent cations with high hydration energy is nonselective [77]. The adsorption of both metal cations reached a plateau after 120 min (Zn^2+^ = 86% and 57%; Cd^2+^ = 53% and 50% for AC and Clin, respectively) due to the saturation of the active sites by MB.

For AC, Zn^2+^ is preferentially adsorbed instead of Cd^2+^. This behavior could be explained by the smaller ionic radius of the Zn cation (0.60–0.90 Å) [78,79]. In fact, it is easier for the Zn cation to diffuse into the mesopores and start the saturation of AC active sites. Moreover, since its ionic radius is smaller than that of Cd cation, more Zn cations can be attracted on the active sites: the smaller the ionic radius, the higher the number of Zn cations on AC active sites. On the other hand, Cd cation has a large ionic radius. Thus, when it diffuses into the AC active sites, these are already partially saturated with Zn and, for this reason, the adsorption of the Cd cation has reached a plateau. Further investigation of the kinetics of these tests will be investigated in Section 3.3.

### 3.3. Abatement Kinetics

To investigate the abatement behavior of Clin and Activated Charcoal (AC) toward Methylene Blue (MB), heavy metals (mainly Zn and Cd), and their combination, different abatement kinetics models are implemented to fit the experimental data, including pseudo-first-order (PFO) (Equation (1)), pseudo-second-order (PSO) (Equation (2)), Elovich (Equation (3)), intraparticle diffusion (Equation (4)), the Bangham model (Equation (5)) and, finally, Avrami kinetic models (Equation (6)) [49,50,51,80,81].

(1) $$\frac{dq}{dt}=k_{1}\cdot \left(q_{e}-q\right)$$

(2) $$\frac{dq}{dt}=k_{2}\cdot \;{\left(q_{e}-q\right)}^{2}$$

(3) $$\frac{dq}{dt}=\alpha \cdot \;exp\left(-\beta \cdot q\right)$$

(4) $$q_{t}=k_{i}\;\cdot \;t^{0.5}+C\;$$

(5) $$ln\left(q_{t}\right)=\vartheta \;\cdot ln\left(t\right)+ln\left(k_{B}\right)$$

(6) $$\frac{dq}{dt}=k\;\cdot n\;\cdot \;t^{n-1}\;\cdot \left(q_{e}-q\right)\;\to \mathrm{with}\;n\;\neq 1$$

The abovementioned models are most commonly used to describe the sorption of dyes as well as other pollutants (heavy metals) on solid sorbents [50].

The kinetics of abatement and the respective parameters were determined by plotting the adsorptive uptake of the dye (or the heavy metals) from the aqueous solution at different time intervals.

For the sake of simplicity, the linearized form was implemented for PFO (Equation (7)), PSO (Equation (8)), Elovich (Equation (9)), and Avrami (Equation (10)) to fit the experimental data as reported in the following equations:

(7) $$ln\left(q_{e}-q_{t}\right)=ln\left(q_{e}\right)-k_{1}\cdot t\;$$

(8) $$\frac{t}{q_{t}}=\frac{1}{k_{2}\;\cdot \;q_{e}^{2}}+\frac{t}{q_{e}}$$

(9) $$q_{t}=\frac{1}{\beta }\;\cdot ln\left(\alpha \cdot \beta \right)+\frac{1}{\beta }\;\cdot ln\left(t\right)\;$$

(10) $$ln\left(ln\left(\frac{q_{e}}{q_{e}-q}\right)\right)=ln\left(k_{A}\right)+n\cdot ln\left(t\right)$$

Starting from the linearization, it is possible to estimate model parameters from the slope and the intercept.

In each model, $q_{t}$ and $q_{e}$ represent the amounts of dye (or heavy metals) adsorbed per gram of adsorbent (both expressed in ${\mathrm{mg}\;g}^{-1}$) at time *t* (min) and at equilibrium, respectively, and calculated from Equation (11) as follows:

(11) $$q=\frac{(C_{0}-C)\;\cdot V}{m}$$

where $C_{0}$ and C  represent the concentration of dye in the bulk liquid at the initial time and time t (both expressed in ${\mathrm{mg}\;L}^{-1}$), respectively, V  is the volume of the bulk solution (L) and m is the mass of adsorbent (g).

The other parameters are $k_{1}$ ($\min^{-1}$), $k_{2}$ (${g\;\mathrm{mg}}^{-1}\min^{-1})$, and $k_{i}$ (${\mathrm{mg}\;g}^{-1}\min^{-0.5})$, which correspond to the rate constants of the PFO, PSO, and intraparticle diffusion models, respectively. Finally, α (${\mathrm{mg}\;g}^{-1}\min^{-1})$, β $\left({g\;\mathrm{mg}}^{-1}\right)$, C $\left({\mathrm{mg}\;g}^{-1}\right)$, $k_{B}$ (${\mathrm{mg}\;g}^{-1}\min^{-1}$), ϑ (-), and $k_{A}$ ($\min^{-n})$, n (-) are specific parameters for each model.

To investigate the effect of the initial concentration of MB on abatement kinetics, different tests were performed by varying the dye concentration from 100 ppm to 250 ppm, and the experimental data were fitted with the aforementioned kinetics models. Finally, the best-fit model was determined based on the determination coefficient value (*R*^2^).

**Figure 7:**
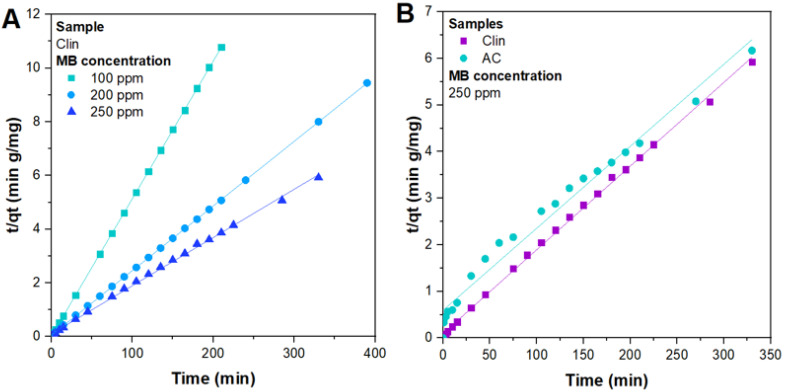
Experimental data of MB abatement tests fitted with a PSO kinetic model with different concentrations of MB on Clin (**A**) and by using 250 ppm MB on Clin and AC (**B**).

For the sake of brevity, only plots with the best fits are reported in Figure 7, whereas the further parameters of the other kinetic models and their respective *R*^2^ coefficients are illustrated in Table 4.

The *pseudo-first-order* equation of Lagergren can be an indication of intraparticle diffusion and/or surface reaction-controlled kinetics [81]. Since *k*_1_ varies with the initial concentration, PFO is representative of surface reaction-controlled kinetics. However, this model proved to be not suitable to fit experimental data, since the determination coefficient *R*^2^ showed a value much lower than unity for each concentration tested.

Furthermore, the experimental and calculated values of equilibrium abatement capacities (*q_e_*) differ from each other for both adsorbents employed, as shown in Table 4 [49,50,80]. This result was consistent with the literature [52,81], where the abatement of MB does not follow a PFO.

The *pseudo-second-order* model assumes that the rate-limiting step may be chemical sorption or chemisorption involving valence forces through the sharing or exchange of electrons between the sorbent (Clin and AC) and sorbate (MB) [50].

According to the fitting, the value of **R*^2^* using the pseudo-second-order kinetic model for Clin and AC showed the highest agreement with the experimental data (*R*^2^ values close to the unity). Moreover, the experimental and calculated amounts of *q_e_* agree with each other, even if sometimes the latter can be lower than the former, as demonstrated in the literature [49,81]. Therefore, the results obtained show the applicability of this kinetic model of the abatement process for each concentration investigated, indicating that the rate of MB abatement process on Clin (or AC) is controlled by chemisorption, as confirmed in the literature [49,80].

It is interesting to highlight that the higher the concentration of MB, the lower the value of $k_{2}$, resulting in a slower kinetic abatement, as illustrated in Figure 4D.

Moreover, comparing the value of $k_{2}$ obtained for Clin and AC at 250 ppm MB, the adsorption was faster for the former than for the latter adsorbent, validating the fact that the abundant presence of cations in the Clin framework is favorable to MB adsorption, as previously explained and illustrated in Figure 5C.

The *Elovich* kinetic model is employed to describe the chemisorption of gases onto heterogeneous surfaces and solid systems, and it is also applied to the study of the removal of pollutants from aqueous solutions [49,52].

The value of α (corresponding to the initial abatement rate), both for Clin and CA for each concentration tested, was observed to be greater than the value of *β* (that is, a desorption constant related to the extent of surface coverage and activation energy for chemisorption), indicating an abatement rate higher than desorption, as confirmed in the literature [49].

However, according to the value of *R*^2^, this model is not suitable to describe the kinetics of abatement of MB using Clin as an adsorbent. In contrast, it is interesting to highlight that the Elovich model is more appropriate to describe the behavior of AC as an adsorbent [81], since it was observed in good accordance with the experimental data, with an *R*^2^ equal to 0.9613, close to that obtained from PSO (0.9786). This means that, in this case, the chemical phenomenon is very important.

The *intraparticle diffusion (IPD)* model is an empirical relationship usually used to describe the rate-determining steps of an abatement process [49,51].

In each test performed, since the parameter ‘*C*’ (which gives an idea of the thickness of the boundary layer [49]) has a value different from zero, the curve does not pass from the origin; therefore, the intraparticle diffusion is not the only control step, and the abatement process is controlled by different steps.

As reported in Table 4, the higher the concentration of MB, the higher the value of *k_i_*. This means that the dye is favored to diffuse inside the adsorbent.

Moreover, a similar trend is observed for coefficient *C*, but with a different magnitude. In this case, a higher value of *C* indicates a greater effect of the boundary layer on the abatement of MB on Clin [49].

On the other hand, using AC as an adsorbent, it was observed that the data were aligned on a straight line, which could be attributed to macropore diffusion [50]. Moreover, *k_i_* presents a higher value than those obtained for Clin, and the ‘*C*’ parameter has a low value. Therefore, MB diffuses easily inside AC due to reduced pore diffusion resistance, as demonstrated from the results obtained from the N_2_ physisorption; thus, the effect of the boundary layer is much lower than that of Clin.

As seen from the value of *R*^2^, this model is not suitable for fitting the experimental data on Clin, but it seems much more appropriate to describe the abatement on AC.

*Bangham*’s model belongs to the internal diffusion model and assumes intraparticle diffusion as the only rate-controlling step. Mainly, this model is used to evaluate the dominance of pore diffusion in the abatement process [82]. In this study, it is not representative of the experimental data obtained on Clin as an adsorbent, since the *R*^2^ is much lower than unity. In contrast, the abatement behavior on AC seems to have good accordance with this model, even if PSO is confirmed to be the best-fitting model.

Sometimes, during the abatement process, it is possible to have an abatement rate coefficient depending on time. In this case, a kinetic system with a time-dependent rate coefficient is said to exhibit “fractal-like kinetics”. In this regard, another kinetic model may be implemented to describe the kinetic behavior, called the *Avrami* rate equation [53,54,81,83]. The n parameter is related to the possible changes in the abatement mechanism that takes place during the abatement process and is a fractional number. Instead of following only an integer-kinetic order, the mechanism abatement could follow multiple kinetic orders that are changed during the contact of the adsorbate with the adsorbent [53].

In this case, the linearization is more complex than the previous models investigated, and for each test performed, it was found to be in poor accordance with the experimental data, with an *R*^2^ coefficient much lower than that obtained for PSO.

**Figure 8:**
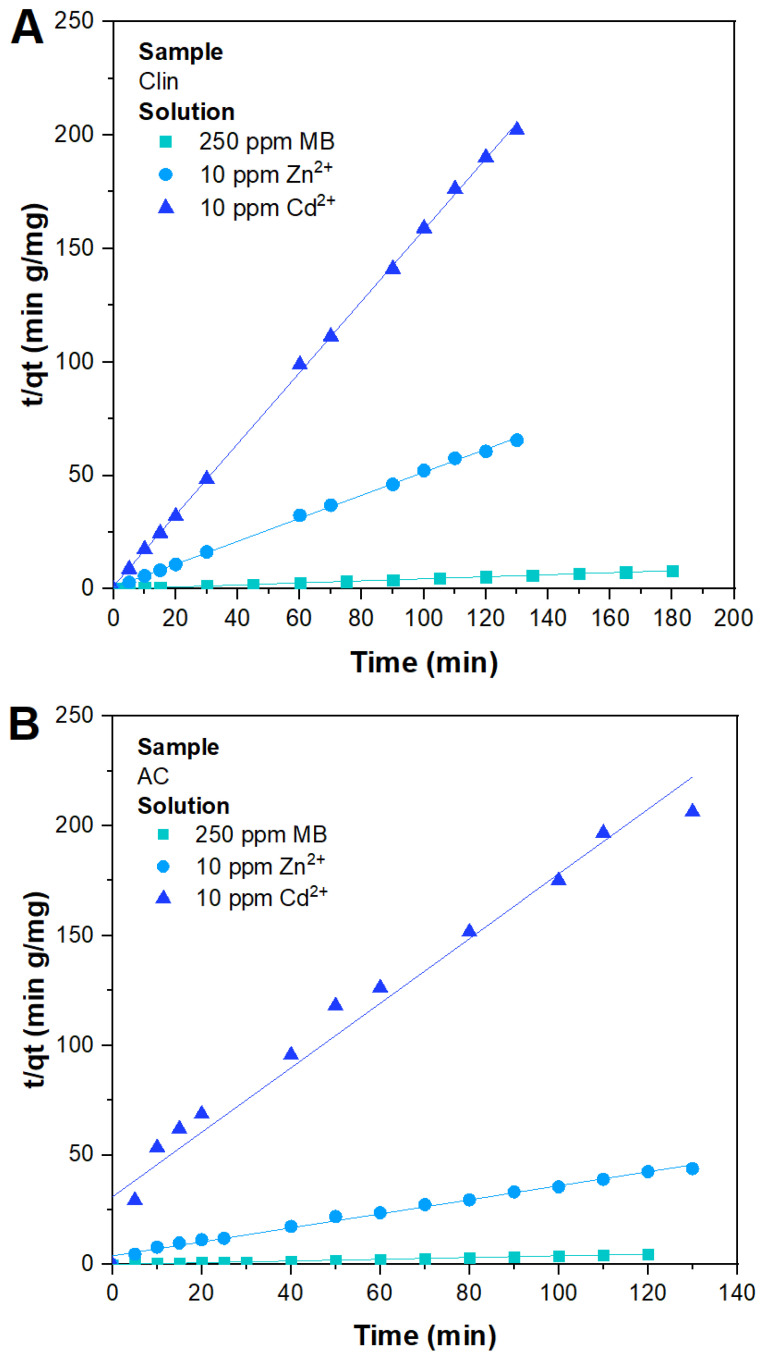
Experimental data of a system with 250 ppm MB, 10 ppm Zn^2+^, and 10 ppm Cd^2+^ fitted with a PSO kinetic model by using Clin (**A**) and AC (**B**) as adsorbents.

Finally, a similar study was conducted to investigate the kinetic behavior of a system with the simultaneous presence of MB and heavy metals, mainly Zn^2+^ and Cd^2+^. As previously, only the best-fitting plot is illustrated (Figure 8), adding further parameters about other models in the summary table (Table 5) reported below.

For the system constituted by the copresence of dye and metals, the abatement kinetics were investigated by applying the analogous model mentioned above. Even in this case, the PSO kinetic model exhibited the best fit over the entire time range for both adsorbents tested, resulting in an *R*^2^ coefficient close to unity and values of $q_{e,fit}$ and $q_{e,exp}$ similar to each other, confirming chemisorption control over the entire abatement process. All the other models investigated are nonrepresentative of the experimental data collected since *R*^2^ showed a value far from unity. As can be observed from the value of $k_{2}$ reported in Table 5, the adsorption rate of MB on Clin was faster with the copresence of dye and metal cations ($k_{2}$ = 0.0609 ${g\;\mathrm{mg}}^{-1}\min^{-1}$) compared to the system in which there was only MB ($k_{2}$ = 0.0031 ${g\;\mathrm{mg}}^{-1}\min^{-1}$). From the results obtained, it seems that the presence of metal cations helps the adsorption of MB on Clin, resulting in faster kinetics.

## 4. Conclusions

In this work, Clinoptilolite, a natural zeolite, was studied for adsorption tests of a probe molecule of organic dyes, Methylene Blue, and for the abatement of metal cations, Zn^2+^ and Cd^2+^. The Activated Charcoal was studied for comparison purposes.

The adsorption tests were performed at different Methylene Blue concentrations with Clinoptilolite, namely 100, 200, and 250 ppm. The best abatement condition was the 100-ppm case study (98%, t = 15 min).

Then, the natural zeolite was compared to Activated Charcoal at 250 ppm of Methylene Blue. It was demonstrated that both materials were very effective in the adsorption of the organic dye since the abatement was approximately 96% at 210 min.

Finally, Clinoptilolite and Activated Charcoal were studied with the copresence of Methylene Blue and specific amounts of Zn^2+^ and Cd^2+^ (10 ppm for each cation). Interestingly, both adsorbents achieved 100% MB abatement (t = 40 min). However, cation adsorption reached a plateau after 120 min (Zn^2+^ = 86% and 57%; Cd^2+^ = 53% and 50% for Activated Charcoal and Clinoptilolite, respectively) due to the preferential adsorption of MB molecules.

From a kinetic point of view, it can be concluded that each case examined follows a pseudo-second-order model, highlighting that a chemisorption process effectively describes the adsorption behavior of MB and heavy metals both on Clinoptilolite and Activated Charcoal.

## Figures and Tables

**Table 1:** Specific Surface Area (SSA), Total Pores Volume (V_TP_), and Total Pores Diameter (D_TP_) of the Clin and AC samples.

Adsorbent Material	SSA (m^2^ g^−1^) ^a^	V_TP_ (cm^3^ g^−1^) ^b^	D_p_
Clin	32	0.12	A channel 3.0 × 7.6 Å ^c^
			B channel 3.3 × 4.6 Å ^c^
			C channel 2.6 × 4.7 Å ^c^
AC	891	0.56	3.3 nm ^d^

^a^ Evaluated by the BET method; ^b^ evaluated by the BJH method during the desorption phase; ^c^ average channel dimensions from [38,47]; ^d^ average pore diameter from [60].

**Table 2:** EDX and XRF results are reported as atomic %.

Adsorbent Material	EDX					XRF				
	Si	Al	K	Ca	Fe	Si	Al	K	Ca	Fe
Clin	72.5	14.9	7.3	3.7	1.6	79.1	11.1	3.9	3.8	2.1

**Table 3:** pH values at the beginning (t = 0 min) and the end (t = 210 min) of MB abatement tests.

Adsorbent Material	MB Concentration (ppm)	pH|_time=0 min_	pH|_time=210 min_
Clin	100	8.36	7.11
	200	8.01	5.65
	250	6.18	6.09

**Table 4:** Kinetic parameters of MB abatement using Clin and AC as adsorbents.

			MB Concentration (ppm)			
			Clin			AC
Model	Equation	Parameters	100	200	250	250
Pseudo-First-Order	(7)	$R^{2}$	0.2496	0.7594	0.9159	0.9188
		$k_{1}$	0.0116	0.0133	0.0111	0.0134
		$q_{e,fit}$	0.7371	6.3630	19.8459	51.4803
		$q_{e,exp}$	19.5897	41.4197	56.2159	53.5020
Pseudo-Second-Order	(8)	$R^{2}$	1	1	0.9985	0.9786
		$k_{2}$	0.4460	0.0127	0.0033	0.0005
		$q_{e,fit}$	19.4932	41.4938	55.5556	56.8182
		$q_{e,exp}$	19.5897	41.4197	56.2159	53.5020
Intraparticle Diffusion	(4)	$R^{2}$	0.3017	0.4887	0.6499	0.9707
		$k_{i}$	0.5235	1.1673	1.9014	3.1658
		C	13.7420	25.6400	28.5310	3.9967
Elovich	(9)	$R^{2}$	0.4478	0.7116	0.8158	0.9613
		α	1693.8155	360.2312	175.6284	6.4746
		β	0.5850	0.2275	0.1559	0.1067
Bangham	(5)	$R^{2}$	0.3179	0.3889	0.4279	0.9158
		ϑ	0.2147	0.2704	0.3063	0.5562
		$k_{B}$	7.4536	11.2211	12.1399	2.8460
Avrami	(10)	$R^{2}$	0.3992	0.7133	0.7088	0.6826
		n	0.2150	0.2959	0.2404	0.4954
		$k_{A}$	1.7049	0.8992	0.7728	0.1353

**Table 5:** Kinetic parameters of the system with 250 ppm MB, 10 ppm Zn^2+^, and 10 ppm Cd^2+^, using Clin and AC as adsorbents.

			$C_{MB}\;$ $=250\;\mathrm{ppm};\;C_{Zn\;}$ $=10\;\mathrm{ppm};\;C_{Cd}=10\;\mathrm{ppm}$					
			Clin			AC		
Model	Equation	Parameters	MB	Zn	Cd	MB	Zn	Cd
Pseudo-First-Order	(7)	$R^{2}$	0.5591	0.2499	0.0434	0.7044	0.6648	0.1891
		$k_{1}$	0.0286	0.0167	0.0067	0.0650	0.0183	0.0086
		$q_{e,fit}$	2.0530	0.3422	0.0576	7.7145	1.7296	0.3836
		$q_{e,exp}$	22.2054	1.9820	0.643	24.4256	2.968	0.6300
Pseudo-Second-Order	(8)	$R^{2}$	0.9999	0.9990	0.9995	0.9979	0.9902	0.9631
		$k_{2}$	0.0609	0.3228	1.8599	0.0108	0.0253	0.0699
		$q_{e,fit}$	22.2717	1.9716	0.6370	25.3807	3.1279	0.6797
		$q_{e,exp}$	22.2054	1.9820	0.6430	24.4256	2.968	0.6300
Intraparticle Diffusion	(4)	$R^{2}$	0.3409	0.4493	0.3961	0.6889	0.9267	0.9844
		$k_{i}$	0.6897	0.0938	0.0289	1.7666	0.2322	0.0537
		C	15.1210	1.0874	0.3732	9.2597	0.5441	0.0353
Elovich	(9)	$R^{2}$	0.4426	0.6557	0.6077	0.8979	0.9920	0.9616
		α	1645.0404	3.6169	1.4994	10.1944	0.5882	0.0832
		β	0.5040	3.4758	11.0011	0.2013	1.6420	7.6746
Bangham	(5)	$R^{2}$	0.3064	0.7467	0.2486	0.7299	0.9356	0.0402
		ϑ	0.2317	0.1050	−0.0474	0.5263	0.2646	0.0721
		$k_{B}$	2.0936	1.2354	0.7519	2.9032	0.8139	0.3194
Avrami	(10)	$R^{2}$	0.4655	0.3438	0.2837	0.5768	0.5305	0.3941
		n	0.2664	0.2064	0.1804	0.5516	0.3342	0.2883
		$k_{A}$	1.4421	1.2376	1.4599	0.4703	0.4316	0.3639

## Data Availability

Not applicable.

## Supplementary Materials

The following supporting information can be downloaded at https://www.mdpi.com/article/10.3390/ma15228191/s1, Figure S1: Methylene Blue adsorption isotherm on Clinoptilolite; Figure S2: Methylene Blue adsorption capacity on Clinoptilolite at different concentrations; Figure S3: Methylene Blue adsorption capacity at 250 ppm on Clinoptilolite and Activated Charcoal; Table S1: QPA results obtained with Clinoptilolite; Table S2: Average costs of Activated Charcoal; Table S3: Comparison of adsorption performance between Clin and other adsorbents in the literature toward different pollutants.

## References

[1] Adhikari B., Palui G., Banerjee A. (2009). Self-Assembling Tripeptide Based Hydrogels and Their Use in Removal of Dyes from Waste-Water. Soft Matter.

[2] McMullan G., Meehan C., Conneely A., Kirby N., Robinson T., Nigam P., Banat I.M., Marchant R., Smyth W.F. (2001). Microbial Decolourisation and Degradation of Textile Dyes. Appl. Microbiol. Biotechnol..

[3] Pearce C.I., Lloyd J.R., Guthrie J.T. (2003). The Removal of Colour from Textile Wastewater Using Whole Bacterial Cells: A Review. Dye. Pigment..

[4] Dosa M., Piumetti M., Galletti C., Russo N., Fino D., Bensaid S., Mancini G., Freyria F.S., Saracco G. (2019). A Novel Fe-Containing Clinoptilolite for Wastewater Remediation: Degradation of Azo-Dyes Acid Orange 7 by H2O2 and Ascorbic Acid. Desalin. Water Treat..

[5] Hussain A., Madan S., Madan R., Nazal M.K., Zhao H. (2021). Removal of Heavy Metals from Wastewater by Adsorption. Heavy Metals.

[6] Łach M., Grela A., Pławecka K., Guigou M.D., Mikuła J., Komar N., Bajda T., Korniejenko K. (2022). Surface Modification of Synthetic Zeolites with Ca and HDTMA Compounds with Determination of Their Phytoavailability and Comparison of CEC and AEC Parameters. Materials.

[7] Piumetti M., Freyria F.S., Armandi M., Geobaldo F., Garrone E., Bonelli B. (2014). Fe- and V-Doped Mesoporous Titania Prepared by Direct Synthesis: Characterization and Role in the Oxidation of AO7 by H2O2 in the Dark. Catal. Today.

[8] Freyria F.S., Compagnoni M., Ditaranto N., Rossetti I., Piumetti M., Ramis G., Bonelli B. (2017). Pure and Fe-Doped Mesoporous Titania Catalyse the Oxidation of Acid Orange 7 by H2O2 under Different Illumination Conditions: Fe Doping Improves Photocatalytic Activity under Simulated Solar Light. Catalysts.

[9] Banat I.M., Nigam P., Singh D., Marchant R. (1996). Microbial decolorization of textile-dye-containing effluents: A review. Bioresour. Technol..

[10] Wang Z., Xue M., Huang K., Liu Z. (2011). Textile Dyeing Wastewater Treatment. Adv. Treat. Text. Effl..

[11] Kumar M.N.V.R., Sridhari T.R., Bhavani K.D., Dutta P.K. (1998). Trends in Color Removal from Textile Mill Effluents. Colourage.

[12] Sun Q., Yang L. (2003). The Adsorption of Basic Dyes from Aqueous Solution on Modified Peat-Resin Particle. Water Res..

[13] Dosa M., Piumetti M., Davarpanah E., Moncaglieri G., Bensaid S., Fino D., Piumetti M., Bensaid S. (2021). Natural Zeolites as Sustainable Materials for Environmental Processes. Nanostructured Catalysts for Environmental Applications.

[14] Dosa M., Piumetti M., Bensaid S., Russo N., Baglieri O., Miglietta F., Fino D. (2018). Properties of the Clinoptilolite: Characterization and Adsorption Tests with Methylene Blue. J. Adv. Catal. Sci. Technol..

[15] Garg V.K., Amita M., Kumar R., Gupta R. (2004). Basic Dye (Methylene Blue) Removal from Simulated Wastewater by Adsorption Using Indian Rosewood Sawdust: A Timber Industry Waste. Dye. Pigment..

[16] Abbas N., Rubab N., Sadiq N., Manzoor S., Khan M.I., Garcia J.F., Aragao I.B., Tariq M., Akhtar Z., Yasmin G. (2020). Aluminum-Doped Cobalt Ferrite as an Efficient Photocatalyst for the Abatement of Methylene Blue. Water.

[17] Clifton J.I.I., Leikin J.B. (2003). Methylene Blue. Am. J. Ther..

[18] Ekambaram S.P., Perumal S.S., Rajendran D., Samivel D., Khan M.N., Bidoia E.D., Montagnolli R.N. (2018). New Approach of Dye Removal in Textile Effluent: A Cost-Effective Management for Cleanup of Toxic Dyes in Textile Effluent by Water Hyacinth. Toxicity and Biodegradation Testing.

[19] EL-Mekkawi D.M., Ibrahim F.A., Selim M.M. (2016). Removal of Methylene Blue from Water Using Zeolites Prepared from Egyptian Kaolins Collected from Different Sources. J. Environ. Chem. Eng..

[20] Mulushewa Z., Dinbore W.T., Ayele Y. (2021). Removal of Methylene Blue from Textile Waste Water Using Kaolin and Zeolite-x Synthesized from Ethiopian Kaolin. Environ. Anal. Health Toxicol..

[21] Galletti C., Dosa M., Russo N., Fino D. (2021). Zn^2+^ and Cd^2+^ Removal from Wastewater Using Clinoptilolite as Adsorbent. Environ. Sci. Pollut. Res..

[22] Mousavi S.A., Shahbazi D., Mahmoudi A., Darvishi P. (2021). Methylene Blue Removal Using Prepared Activated Carbon from Grape Wood Wastes: Adsorption Process Analysis and Modeling. Water Qual. Res. J..

[23] Nicola R., Muntean S.-G., Nistor M.-A., Putz A.-M., Almásy L., Săcărescu L. (2020). Highly Efficient and Fast Removal of Colored Pollutants from Single and Binary Systems, Using Magnetic Mesoporous Silica. Chemosphere.

[24] Oves M., Saghir Khan M., Huda Qari A., Nadeen Felemban M., Almeelbi T. (2016). Heavy Metals: Biological Importance and Detoxification Strategies. J. Bioremediat. Biodegrad..

[25] Fu Z., Xi S. (2020). The Effects of Heavy Metals on Human Metabolism. Toxicol. Mech. Methods.

[26] Srivastava N.K., Majumder C.B. (2008). Novel Biofiltration Methods for the Treatment of Heavy Metals from Industrial Wastewater. J. Hazard. Mater..

[27] (2013). Us Epa National Primary Drinking Water Regulations. Drink. Water Contam..

[28] Bedrin A.G., Bubnov I.A., Dashuk S.P., Mironov I.S. (2003). Compact Spectral Analyzer of Heavy-Metal Impurities in Air. J. Opt. Technol..

[29] World Health Organization (WHO) (2004). Guidelines for Drinking-Water Quality.

[30] EPA (2004). Technical Support Document for the 2004 Effluent Guidelines Program Plan.

[31] Gazzetta Ufficiale della Repubblica Italiana, D.Lgs 152/2006—Norme in Materia Ambientale. https://www.gazzettaufficiale.it/dettaglio/codici/materiaAmbientale.

[32] O’Connell D.W., Birkinshaw C., O’Dwyer T.F. (2008). Heavy Metal Adsorbents Prepared from the Modification of Cellulose: A Review. Bioresour. Technol..

[33] Hunsom M., Pruksathorn K., Damronglerd S., Vergnes H., Duverneuil P. (2005). Electrochemical Treatment of Heavy Metals (Cu^2+^, Cr^6+^, Ni^2+^) from Industrial Effluent and Modeling of Copper Reduction. Water Res..

[34] Crini G., Lichtfouse E. (2019). Advantages and Disadvantages of Techniques Used for Wastewater Treatment. Environ. Chem. Lett..

[35] Zhang L., Wu Y., Qu X., Li Z., Ni J. (2009). Mechanism of Combination Membrane and Electro-Winning Process on Treatment and Remediation of Cu^2+^ Polluted Water Body. J. Environ. Sci..

[36] Bobade V., Eshtiaghi N. (2015). Heavy Metals Removal from Wastewater by Adsorption Process: A Review.

[37] Inglezakis V.J., Zorpas A.A. (2012). Handbook of Natural Zeolites.

[38] Kennedy D., Tezel F.H. (2018). Cation Exchange Modification of Clinoptilolite—Screening Analysis for Potential Equilibrium and Kinetic Adsorption Separations Involving Methane, Nitrogen, and Carbon Dioxide. Microporous Mesoporous Mater..

[39] Mumpton F.A. (1960). Clinoptilolite Redefined. Am. Mineral..

[40] Mumpton F.A. (1999). La Roca Magica: Uses of Natural Zeolites in Agriculture and Industry. Proc. Natl. Acad. Sci. USA.

[41] Tsitsishvili G., Andronikashvili T., Kirov G., Filizova L. (1992). Natural Zeolites.

[42] Osmanlioglu A.E. (2006). Treatment of Radioactive Liquid Waste by Sorption on Natural Zeolite in Turkey. J. Hazard. Mater..

[43] Cooney E.L., Booker N.A., Shallcross D.C., Stevens G.W. (1999). Ammonia Removal from Wastewaters Using Natural Australian Zeolite. I. Characterization of the Zeolite. Sep. Sci. Technol..

[44] Blanchard G., Maunaye M., Martin G. (1984). Removal of Heavy Metals from Waters by Means of Natural Zeolites. Water Res..

[45] Moirou A., Xenidis A., Paspaliaris I. (2001). Stabilization Pb, Zn, and Cd-Contaminated Soil By Means of Natural Zeolite. Soil Sediment Contam. Int. J..

[46] Armbruster T., Galarneau A., Fajula F., di Renzo F., Vedrine J. (2001). Clinoptilotite-Heulandite: Applications and Basic Research. Studies in Surface Science and Catalysis.

[47] Davarpanah E., Armandi M., Hernández S., Fino D., Arletti R., Bensaid S., Piumetti M. (2020). CO_2_ Capture on Natural Zeolite Clinoptilolite: Effect of Temperature and Role of the Adsorption Sites. J. Environ. Manag..

[48] Jayaraman A., Hernandez-Maldonado A.J., Yang R.T., Chinn D., Munson C.L., Mohr D.H. (2004). Clinoptilolites for Nitrogen/Methane Separation. Chem. Eng. Sci..

[49] Noori M., Tahmasebpoor M., Foroutan R. (2022). Enhanced Adsorption Capacity of Low-Cost Magnetic Clinoptilolite Powders/Beads for the Effective Removal of Methylene Blue: Adsorption and Desorption Studies. Mater. Chem. Phys..

[50] Aysan H., Edebali S., Ozdemir C., Celiїk Karakaya M., Karakaya N. (2016). Use of Chabazite, a Naturally, Abundant Zeolite, for the Investigation of the Adsorption Kinetics and Mechanism of Methylene Blue Dye. Microporous Mesoporous Mater..

[51] Largitte L., Pasquier R. (2016). A Review of the Kinetics Adsorption Models and Their Application to the Adsorption of Lead by an Activated Carbon. Chem. Eng. Res. Des..

[52] Varank G., Demir A., Yetilmezsoy K., Top S., Sekman E., Bilgili S. (2012). Removal of 4-Nitrophenol from Aqueous Solution by Natural Low-Cost Adsorbents. Indian J. Chem. Technol..

[53] Cardoso N.F., Pinto R.B., Lima E.C., Calvete T., Amavisca C.V., Royer B., Cunha M.L., Fernandes T.H.M., Pinto I.S. (2011). Removal of Remazol Black B Textile Dye from Aqueous Solution by Adsorption. Desalination.

[54] Oladoja N.A. (2016). A Critical Review of the Applicability of Avrami Fractional Kinetic Equation in Adsorption-Based Water Treatment Studies. Desalin. Water Treat..

[55] Ho Y.S., McKay G. (2003). Sorption of Dyes and Copper Ions onto Biosorbents. Process Biochem..

[56] Munir M., Nazar M.F., Zafar M.N., Zubair M., Ashfaq M., Hosseini-Bandegharaei A., Khan S.U.D., Ahmad A. (2020). Effective Adsorptive Removal of Methylene Blue from Water by Didodecyldimethylammonium Bromide-Modified Brown Clay. ACS Omega.

[57] Bayomie O.S., Kandeel H., Shoeib T., Yang H., Youssef N., El-Sayed M.M.H. (2020). Novel Approach for Effective Removal of Methylene Blue Dye from Water Using Fava Bean Peel Waste. Sci. Rep..

[58] Almaamary E.A.S., Abdullah S.R.S., Hasan H.A., Rahim R.A.A., Idris M. (2017). Rawatan Metilena Biru Dalam Air Sisa Menggunakan Scirpus Grossus. Malays. J. Anal. Sci..

[59] Ahmed T., Noor W., Faruk O., Bhoumick M.C., Uddin M.T. (2018). Removal of Methylene Blue (MB) from Waste Water by Adsorption on Jackfruit Leaf Powder (JLP) in Continuously Stirred Tank Reactor. Journal of Physics: Conference Series.

[60] Ustinov E.A., Do D.D. (2006). Invited Contribution Adsorption in Slit Pores and Pore-Size Distribution: A Molecular Layer Structure Theory. Adsorpt. Sci. Technol..

[61] Arcoya A., González J.A., Travieso N., Seoane X.L. (1994). Physicochemical and Catalytic Properties of a Modified Natural Clinoptilolite. Clay Min..

[62] Liu X.Y., Huang M., Ma H.L., Zhang Z.Q., Gao J.M., Zhu Y.L., Han X.J., Guo X.Y. (2010). Preparation of a Carbon-Based Solid Acid Catalyst by Sulfonating Activated Carbon in a Chemical Reduction Process. Molecules.

[63] Khalil H.P.S., Jawaid M., Firoozian P., Rashid U., Islam A., Akil H.M. (2013). Activated Carbon from Various Agricultural Wastes by Chemical Activation with KOH: Preparation and Characterization. J. Biobased Mater. Bioenergy.

[64] Mumpton F.A., Ormsby W.C. (1976). Morphology of Zeolites in Sedimentary Rocks by Scanning Electron Microscopy. Clays Clay Min..

[65] Abramoff M., Magalhães P., Ram S.J. (2003). Image Processing with ImageJ. Biophotonics Int..

[66] Kannan N., Sundaram M.M. (2001). Kinetics and Mechanism of Removal of Methylene Blue by Adsorption on Various Carbons—A Comparative Study. Dye. Pigment..

[67] Badeenezhad A., Azhdarpoor A., Bahrami S., Yousefinejad S. (2019). Removal of Methylene Blue Dye from Aqueous Solutions by Natural Clinoptilolite and Clinoptilolite Modified by Iron Oxide Nanoparticles. Mol. Simul..

[68] Ćurković L., Cerjan-Stefanović Š., Filipan T. (1997). Metal Ion Exchange by Natural and Modified Zeolites. Water Res..

[69] Leppert D. (1990). Heavy Metal Sorption with Clinoptilolite Zeolite. Alternatives for Treating Contaminated Soil and Water. Min. Eng..

[70] Kesraoui-Ouki S., Cheeseman C.R., Perry R. (1994). Natural Zeolite Utilisation in Pollution Control: A Review of Applications to Metals’ Effluents. J. Chem. Technol. Biotechnol. Int. Res. Process Environ. Clean Technol..

[71] Rafatullah M., Sulaiman O., Hashim R., Ahmad A. (2010). Adsorption of Methylene Blue on Low-Cost Adsorbents: A Review. J. Hazard. Mater..

[72] León M., Silva J., Carrasco S., Barrientos N. (2020). Design, Cost Estimation and Sensitivity Analysis for a Production Process of Activated Carbon from Waste Nutshells by Physical Activation. Processes.

[73] Ng C., Marshall W.E., Rao R.M., Bansode R.R., Losso J.N. (2003). Activated Carbon from Pecan Shell: Process Description and Economic Analysis. Ind. Crops Prod..

[74] Lima I.M., McAloon A., Boateng A.A. (2008). Activated Carbon from Broiler Litter: Process Description and Cost of Production. Biomass Bioenergy.

[75] Stavropoulos G.G., Zabaniotou A.A. (2009). Minimizing Activated Carbons Production Cost. Fuel Process. Technol..

[76] Figueiredo J.L., Pereira M.F.R. (2010). The Role of Surface Chemistry in Catalysis with Carbons. Catal. Today.

[77] Colella C., Misaelides P., Macášek F., Pinnavaia T.J., Colella C. (1999). Environmental Applications of Natural Zeolitic Materials Based on Their Ion Exchange Properties. Natural Microporous Materials in Environmental Technology.

[78] Pauling L. (1992). The Nature of the Chemical Bond—1992. J. Chem. Educ..

[79] Ahrens L.H. (1952). The Use of Ionization Potentials Part 1. Ionic Radii of the Elements. Geochim. Cosmochim. Acta.

[80] Alpat S.K., Özbayrak Ö., Alpat Ş., Akçay H. (2008). The Adsorption Kinetics and Removal of Cationic Dye, Toluidine Blue O, from Aqueous Solution with Turkish Zeolite. J. Hazard. Mater..

[81] Tan K.L., Hameed B.H. (2017). Insight into the Adsorption Kinetics Models for the Removal of Contaminants from Aqueous Solutions. J. Taiwan Inst. Chem. Eng..

[82] Edet U.A., Ifelebuegu A.O. (2020). Kinetics, Isotherms, and Thermodynamic Modeling of the Adsorption of Phosphates from Model Wastewater Using Recycled Brick Waste. Processes.

[83] Songolzadeh M., Soleimani M., Takht Ravanchi M. (2015). Using Modified Avrami Kinetic and Two Component Isotherm Equation for Modeling of CO2/N2 Adsorption over a 13X Zeolite Bed. J. Nat. Gas Sci. Eng..

