# Supplementary material for: Natural Zeolite Clinoptilolite Application in Wastewater Treatment: Methylene Blue, Zinc and Cadmium Abatement Tests and Kinetic Studies
Source: Materials (Basel). 2022 Nov 18;15(22):8191. doi: 10.3390/ma15228191 (PMC9695311; doi:10.3390/ma15228191)
Supplement: Supplementary file 1 [file materials-15-08191-s001.zip › materials-1976450-supplementary.pdf]

Supporting Information

Natural zeolite Clinoptilolite application in wastewater treatment: Methylene Blue, Zinc and Cadmium abatement tests and kinetic studies.

Melodj Dosa [1], Nadia Grifasi [1], Camilla Galletti [1], Debora Fino [1]

and Marco Piumetti [1,]*

1. Department of Applied Science and Technology (DISAT), Politecnico di Torino

Corso Duca degli Abruzzi 24, 10129, Turin, Italy.

* Corresponding Author:

E-mail: marco.piumetti@polito.it

Index

Figure S1. Methylene Blue adsorption isotherm on Clinoptilolite.

Figure S2. Methylene Blue adsorption capacity on Clinoptilolite at different concentrations.

Figure S3. Methylene Blue adsorption capacity at 250 ppm on Clinoptilolite and Activated Charcoal.

Table S1. QPA results obtained with Clin.

Table S2. Average costs of Activated Charcoal.

Table S3. Comparison of adsorption performance between Clin and other adsorbents in literature toward different pollutants.

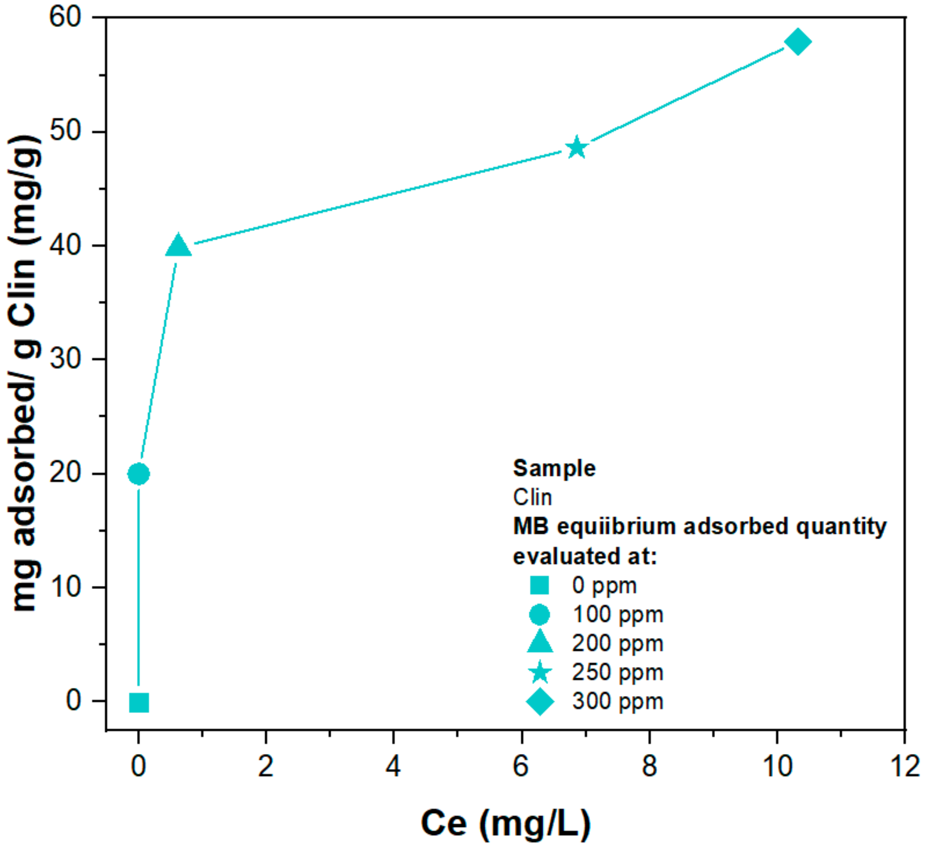

Figure S1. Methylene Blue adsorption isotherm on Clinoptilolite.

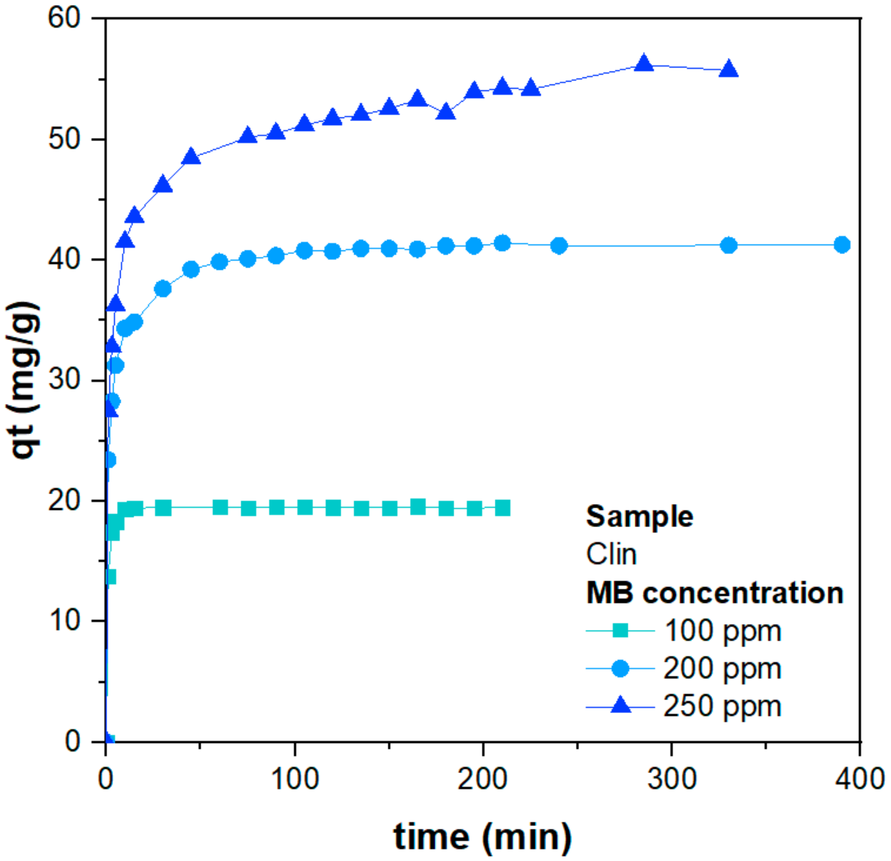

Figure S2. Methylene Blue adsorption capacity on Clinoptilolite at different concentrations.

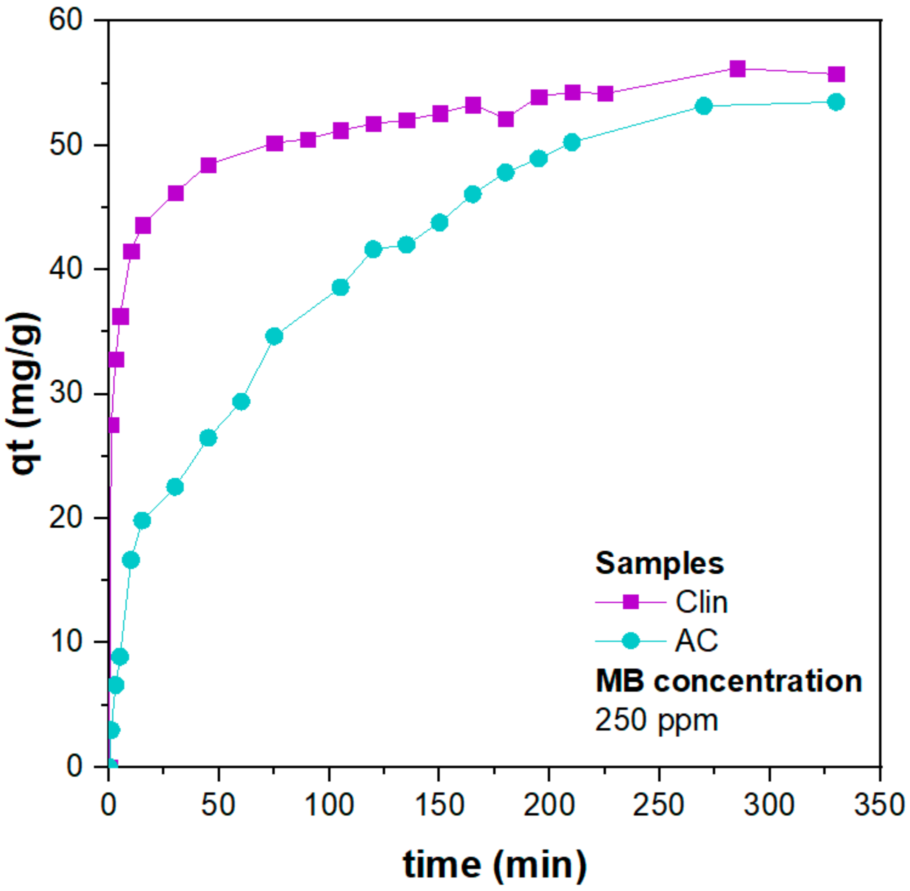

Figure S3. Methylene Blue adsorption capacity at 250 ppm on Clinoptilolite and Activated Charcoal.

Table S1. QPA results obtained with Clinoptilolite.

**Adsorbent material: Clin**		
**QPA results (wt.%)**	Clinoptilolite	66.4 (1)
	Illite	0.8 (2)
	Kaolinite	4.0 (2)
	Amorphous phase	28.8 (1)
**Refinement statistic**	R_{wp} (%)	0.089
	Rp (%)	0.059
	RF^2 (%)	0.086

Table S2. Average costs of Activated Charcoal [72].

Matrix	Cost (USD kg^{-1})	Reference
Pecan shell	2.72-2.89	Ng et al. [73]
Poultry waste	1.44	Lima et al. [74]
Tires	2.23	Stavropoulos et al. [75]
Wood,	2.49	
Petroleum coke	1.08	
Carbon black	1.22	
Coal	1.25	
Lignite	2.18	

Table S3. Comparison of adsorption performance between Clin and other adsorbents in literature toward different pollutants.

Adsorbent	Pollutant	Initial concentrations of the dyes	Abatement	Time	pH	Adsorbed dye over mass of adsorbent (mg/g)	Comments[a]	Ref.
Clin	MB	100 ppm<br>200 ppm<br>250 ppm	100%<br>99%<br>93%	Few min<br>210 min<br>210 min	6-7	19.49<br>41.3<br>55.74	5 g/L of Clin	This work
Clin	MB<br>Zn<br>Cd	250 ppm MB<br>10 ppm Zn<br>10 ppm Cd	99.9%<br>57%<br>51%	130 min	6-7	22.21<br>1.982<br>0.643	10 g/L of Clin	This work
Peptide (hydrogel)	Rhodamine B<br>Reactive blue 4<br>Direct red 80	0.479 mg/mL<br>0.637 mg/mL<br>1.373 mg/mL	83.9%<br>87.9%<br>97.2%	28h<br>30h<br>32h	11.5 - 13.5	7.36<br>9.8<br>10.56	-	1
Fe-Clin	AO7	1.42 mM	60%	10 min	-	-	100 ml AO7 (1.42 mM)+0.1 g Fe-Clin+AA(2.67mM)	4
Fe-MT<br>Fe-IT<br>V-MT<br>MT<br>V-IT<br>P25	AO7	1 g/L	90%<br>80%<br>60%<br>60%<br>30%<br>20%	96 h	-	-	0.1 g/L of catalysts + 0.8 M of H_2O_2	7
MT<br>P25<br>Fe2.5-MTd<br>Fe0.8-IT	AO7	1 g/L	66%<br>25%<br>90%<br>98%	96 h	6.8	-	0.8M H_2O_2	8

Fe2.5-MTi<br>Fe2.5-IT			100%<br>79%					
Clin	Zn<br>Cd	10 g/L	98%<br>100%	180 min<br>Few min	4.5	1<br>0,97	C_{Clin} =10 g/L with 10 mg/L of metal	9
Peat-resin particle	Basic Magenta	200 g/L	n. g. *	700 min	-	40		13
Clin	MB	250 mg/L<br>500 mg/L	96%<br>92.5%	130 min<br>120 min	-	24<br>46.2	50 mg/L of MB with 5 g of Clin	15
Indian Rosewood sawdust	MB	50 g/L	99.9%	120 min				16
$Co_{0.1}Al_{0.03}Fe_{0.17}O_{0.4}$	MB	10 mg/L	83%	120 min	11		0.5 g/L of adsorbent	17
Zeolite ZK<br>ZD<br>ZS	MB	10 mg/L	97%<br>95%<br>92%	120 min	7	21.4<br>13.49<br>11.13	100 ml dye solution with 0.1 g of zeolite	20
Elecopper 25A	Cu	7.84 mol/m^3	100%	4 h	1	-	Electrolysis j= 10 A/m2 0.15 vol% brightener	33
	Cu + Cr	7.84 +10.06 mol/ m^3	96%Cu (<10%Cr)	7 h				
	Cu+ Ni	7.84 + 8.26 mol/m3	<10%Cu (<10%Ni)	7 h				

[a] Temperature: RT. * Not given.
